# Meta‐Analysis of *CYP1A1* MspI and Ile462Val Polymorphisms in Cancer Susceptibility Among Different Ethnic Populations

**DOI:** 10.1002/em.70042

**Published:** 2025-12-10

**Authors:** Amjad Yousuf, Najeeb Ullah Khan, Ahsanullah Unar

**Affiliations:** ^1^ Clinical Laboratory Sciences Department College of Applied Medical Sciences, Taibah University Madinah Saudi Arabia; ^2^ Institute of Biotechnology and Genetic Engineering The University of Agriculture Peshawar Pakistan; ^3^ Department of Precision Medicine University of Campania ‘L. Vanvitelli’ Naples Italy

**Keywords:** cancer risk, cancer susceptibility, CYP1A1 mutation, ethnicity, genetic association, Ile462Val polymorphism, MspI

## Abstract

The Cytochrome P450 1A1 (*CYP1A1*) gene plays a crucial role in the production of enzymes involved in the metabolic activation and detoxification of harmful carcinogens, which are essential for genetic susceptibility to cancer. Due to the inconsistent findings obtained from population‐based studies, it is crucial to systematically investigate the association between *CYP1A1* polymorphisms and diverse ethnic groups. To assess the link between *CYP1A1* polymorphisms and cancer risk across different ethnic populations. The studies published in the last decade were searched through PubMed, Cochrane Library, and Embase, based on PRISMA guidelines and eligibility criteria. Meta‐analysis includes subgroup analysis based on ethnicity with odds ratio (OR) and 95% confidence intervals through R Studio. Genotypic and allelic data were analyzed under genetic models (allelic, dominant, and recessive) using a random‐effects model. The quality of the included case–control studies was assessed using the Newcastle‐Ottawa scale. Twenty case–control studies containing various ethnic populations, of which eleven contain the MspI polymorphism, and the other nine contain the Ile462Val polymorphism of the *CYP1A1*, while none explained both SNPs. The research studies involved 3976 cases and 4891 controls in this meta‐analysis. For MspI polymorphisms, the overall pooled analysis revealed a significant association with cancer risk in the Brazilian ethnic group (2.46 [95% CI: 0.00; 305699178.1]) with moderate heterogeneity observed within the genetic models of *CYP1A1* polymorphisms. For Ile462Val polymorphisms, the overall pooled effect size was significant among the Asian group (2.11 [95% CI: 1.45; 3.06]). Meanwhile, the subgroup analysis provides some evidence of cancer risk association with polymorphisms among different ethnicities. The results of this meta‐analysis indicate that the understanding of *CYP1A1* polymorphisms is necessary to determine the etiology of cancer. The significant association among *CYP1A1* polymorphisms and cancer can further be studied by selecting studies focused on a particular cancer type and containing a large sample size within a specific ethnic population.

## 1 Introduction

Cancer remains a formidable global health challenge because of its substantial increase in incidence and mortality rates (McGee and Nichols 2016). According to global cancer statistics, there were an estimated 20 million new cases of cancer diagnosed, and 9.7 million deaths related to cancer were reported (Bray et al. 2024). Cancer is a multifactorial disease that can result from the influence of environmental factors or the accumulation of mutations that deregulate cellular differentiation, proliferation, and survival rates among individuals. Genetic predisposition to disease is considered crucial in modulating the individual's susceptibility to various cancer types across different ethnic populations. Genetic factors can influence carcinogen metabolism, DNA repair mechanisms, and immune responses, impacting increased cancer susceptibility among individuals. Given the anticipated genetic susceptibility, it is important to comprehend the association of genetic mutations and their polymorphisms with the risk of developing cancer, with the advancements in sequencing and genotyping methods (Manchanda and Sideris 2023; Zhu et al. 2016).

Cytochrome P450 1A1 (*CYP1A1*) gene is present on chromosome 15q22‐q24, spans 5987 base pairs, and encodes a 512 amino acid protein, which produces an enzyme that metabolizes estrogen, producing 2‐hydroxy and 4‐hydroxy estrogen metabolites (Crofts et al. 1993; Sowers et al. 2006). The *CYP1A1* gene encodes the cytochrome P450 superfamily of enzymes involved in drug metabolism and oxidative metabolism of xenobiotics, including polycyclic aromatic hydrocarbons (PAHs), heterocyclic amines, and other environmental pro‐carcinogens (Crofts et al. 1993; Guengerich and Shimada 1998; Li et al. 2016). Therefore, the enzymatic activity of *CYP1A1* is involved in both bio‐activation and detoxification of harmful components. Normally, the hydroxylation process is carried out by *CYP1A1* during the first stage of estrogen metabolism (Guengerich and Shimada 1998; Khvostova et al. 2012). Moreover, it is also involved in the conversion of estrogen to 2‐hydroxy catechol metabolites and 2‐hydroxylation of estradiol for O‐methylation to 2‐methoxy conversion (Michnovicz and Rosenberg 1992). The investigation of *CYP1A1* polymorphisms is necessary to find out its association with hormone‐related cancer susceptibility (Aktas et al. 2002; Arvanitis et al. 2003; Napoli et al. 2005). The alterations in *CYP1A1* expression and function can disrupt the balance between metabolic activation and detoxification, potentially leading to DNA damage and increased cancer risk (Kleine et al. 2015; Li et al. 2016; Proença et al. 2015).

*CYP1A1* exhibits several polymorphisms that have been identified by researchers and found to be associated with the risk of developing various cancer types each with specific nucleotide changes that can affect gene expression or enzyme activity (Amrani et al. 2016; Tan et al. 2016; Zhao et al. 2021). A *CYP1A1* polymorph refers to the different allelic variations or forms of the *CYP1A1* gene in a population. The most commonly studied polymorphism of *CYP1A1* is the MspI (3801TNC, m1, or rs46466903) polymorphism, which is characterized by a T to C at nucleotide 3801 in the 30 flanking region of the *CYP1A1* gene (Oyama et al. 1995). While the Ile462Val (m2 or rs1048943) polymorphism results in the transition of A to G at codon 462 in exon 7, resulting in the substitution of amino acids isoleucine with valine, increasing enzyme activity (Oyama et al. 1995). Several studies have investigated the *CYP1A1* polymorphism in cancer susceptibility among different ethnic populations (Elderdery et al. 2024; Kleine et al. 2015; Li et al. 2016; Proença et al. 2015; Xu and Tan 2024). The distribution of genotypes and allelic variants exhibits significant variability that may contribute to the differences in genetic susceptibility across populations. Numerous case–control studies investigated the *CYP1A1* polymorphism and various cancer types, while the results of genetic and allelic distribution among ethnic populations are inconsistent due to limited sample size, high heterogeneity, and lack of ethnic stratification.

The inconsistent findings of individual studies finding the association between *CYP1A1* polymorphisms and cancer risk or susceptibility underscore the need for a more robust and comprehensive analysis. Therefore, meta‐analysis is performed to gather the systematic pooled effect size data from individual studies to derive a significant association between *CYP1A1* polymorphisms and cancer risk. The meta‐analysis approach for this research makes it different from previous meta‐analyses in that it solely considers studies from the last 10 years, performs subgroup analyses over four continental groups, and assesses publication bias through the application of both Begg's and Egger's tests. In population‐based studies of genetic polymorphisms and cancer risk, a stratified analysis is essential to understand and interpret the associations within case–control study designs because of different genetic backgrounds and environmental exposures across populations. The primary objectives of this study are to perform a substantial population‐based systematic review and meta‐analysis to assess the overall association between *CYP1A1* (MspI and Ile462Val) polymorphisms and various cancer types. While the secondary objectives include implementing subgroup analysis based on ethnicity, evaluating the heterogeneity among the included studies, and assessing the presence of publication bias. By addressing these objectives, this study sought to validate the role of *CYP1A1* genetic variants in cancer development and progression, which can be useful as biomarkers for early cancer diagnosis, control, and treatment.

## 2 Methods

### 2.1 Study Protocol

The design of this study implied guidelines defined by the Preferred Reporting Items for Systematic Review and Meta‐Analysis (PRISMA) for the selection and identification of case–control studies to perform a systematic review and meta‐analysis (Page et al. 2021).

### 2.2 Search Strategy

To determine the association between *CYP1A1* polymorphisms and cancer susceptibility, it is advisable to first identify, select, and gather relevant data from the literature. For this, a well‐defined and systematic search strategy was performed for the identification and selection of eligible research studies that comprehend the *CYP1A1* polymorphisms association with cancer risk. To design an efficient search strategy, it is important to integrate MeSH terms, subject headings, and suitable keywords related to the research question and objectives of this meta‐analysis. To identify the potential research studies comprehending the association of CYP1A1 polymorphisms and cancer risk among diverse ethnic populations, keywords like “CYP1A1,” “cytochrome p‐450 cyp1a1,” “polymorphisms”, “genetic mutation,” “variants,” “MspI,” “Ile462Val,” “cancer risk,” and “susceptibility,” were utilized in the PubMed database. Moreover, several other terms and keywords can also be included that follow a specific study design such as “case–control” and “genome‐wide association studies or GWAS”. These MeSH terms and keywords were combined using Boolean operators like AND, OR, and NOT to specify the search criteria for the identification of the relevant research studies. Following the identification of studies that appear in PubMed, several filters were employed for an efficient screening of the research studies. The filters like publication date, abstract, full‐text, free full‐text, English language, and experiments performed on humans sort out the studies based on their availability and quality, while the remaining studies that do not fulfill the criteria of screening were excluded. At last, the remaining studies were assessed for eligibility based on the inclusion and exclusion criteria of this study. The final studies that are found to be highly qualified and relevant to the design of this study are included in the meta‐analysis.

### 2.3 Inclusion and Exclusion Criteria

Eligibility criteria were defined to specify which studies can be included and which can be excluded, which is most probably characterized based on the PICO framework. During the screening of the research studies, two reviewers independently perform title and abstract screening to avoid any inconsistent results. In this study, the inclusion criteria involved the population of any age, gender, and ethnic background exhibiting *CYP1A1* mutation, reporting genotype distribution of *CYP1A1* polymorphisms or SNPs among cancer individuals. No specific intervention is particularly studied, but involved those studies examining major *CYP1A1* polymorphisms (MspI and Ile462Val), while data requirements must include genotypic and allelic distribution of *CYP1A1* polymorphisms among case and control groups. Case–control studies were highly recommended. The inclusion criteria also prefer those studies that were published in the last 10 years. Meanwhile, the exclusion criteria of studies focused on cancer progression, survival, or response to any treatment rather than cancer susceptibility. Studies on cancer progression, survival or treatment response were excluded as the primary objective of this meta‐analysis was to evaluate the association between *CYP1A1* polymorphisms and cancer susceptibility, which is the genetic predisposition to develop cancer, not the clinical course or treatment outcome. Including such studies would introduce a lot of heterogeneity and deviate from our research question. Studies that involved inadequately defined ethnic classification of individuals and insufficiently duplicated or overlapped data among populations can also be excluded. The studies that exhibit a study design other than case–control or genome‐wide association studies, like case reports, review articles, letters, protocols, meta‐analysis, animal, and in vitro studies, were also removed.

### 2.4 Data Extraction

The data extraction process involved the collection of information or statistical data in one place from all of the final included studies by formulating a standard data extraction form or table. This data extraction form or table must include information like (author, publication year of the study, or study country), characteristics of the study (design, population/ethnicity, or sample size), characteristics of the participants (age, gender, demographics, or type of cancer), *CYP1A1* mutation (specific genotype polymorphs or genotyping method), and statistical data (genotype and allelic distribution among case and control groups) required to perform meta‐analysis. The *CYP1A1* polymorphs were sorted out among case and control groups as wild, heterozygote, and mutant for genotype distribution and wild or mutant allele for allelic distribution of individuals. This data is basically in the form of numbers and percentages necessary for further implementation in association analysis to calculate the odds ratio among different ethnic populations.

### 2.5 Quality Assessment

The quality of the included studies was assessed by two reviewers independently, and any disagreements between the reviewers will be resolved by a thorough discussion or the involvement of a third reviewer if needed. In case of case–control or association studies, quality assessment was probably done using the Newcastle Ottawa Scale (NOS), containing a total score from 0 to 9. Each study was efficiently evaluated based on the selection or definition of case/controls, comparability, and outcome domain among the research studies. Each study was extensively assessed for its quality within the above‐stated domains and assigned probable stars. The total NOS score ranges determine the quality of the included studies.

### 2.6 Statistical Analysis

A statistical meta‐analysis was conducted through R Studio software (2024.09.1, Build +394), where the effect size for the genetic and allelic distribution among cancer individuals was calculated as an odds ratio (OR) with a corresponding 95% confidence interval (95% CI). For this, the outcome data extracted from the studies include the total number (*n*, %) of genotype distribution (wild, heterozygote, or mutant) among case and control groups, with a total sample of participants assigned to each group. A random‐effects model was employed to calculate the pooled effect size with a significant *p*‐value (< 0.005). For heterogeneity, *I* square test statistics were used to quantify the percentage of the total variations across studies, whose value greater than 50% was considered indicative of substantial heterogeneity. Publication bias was assessed through Egger's linear regression and Begg's rank correlation tests within genetic models of *CYP1A1* polymorphisms. Genetic inheritance models were analyzed for MspI and Ile462Val polymorphisms, including allelic, dominant, and recessive models. A subgroup analysis was performed based on ethnicity (Caucasian, Brazilian, Asian, and others). Pooled effect size and publication bias were evaluated using forest and funnel plots, respectively. The forest plot indicates the effect size of each included study in the form of a square box, while the overall or pooled effect size of all studies appears as a diamond with a line of no effect (dotted line). The funnel plot displays the effect size of individual studies against a measure of their precision (standard error), suggesting the presence of publication bias due to its asymmetry. By interpreting the results of forest and funnel plots, the significant association between *CYP1A1* SNPs and cancer susceptibility can be efficiently evaluated.

### 2.7 Hardy Weinberg Equilibrium (HWE) Assessment

While HWE assessment is important for control populations, individual study data on HWE was not available for all included studies and therefore HWE was not formally assessed for each study in this meta‐analysis. This is considered a major limitation of this meta‐analysis and future analyses must include studies that report HWE or calculate it when genotype data is provided.

## 3 Results

### 3.1 Selection of Eligible Studies

**FIGURE 1:**
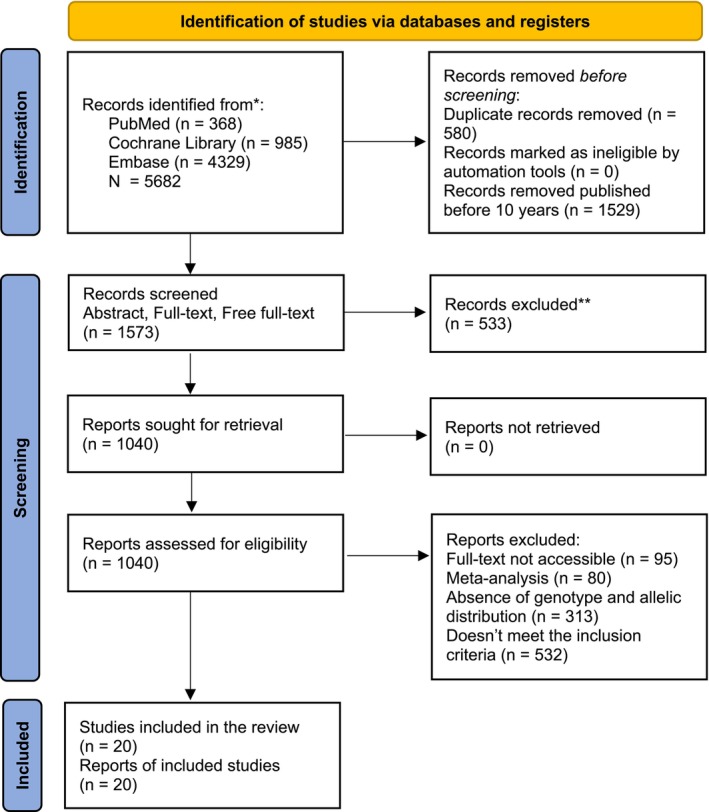
Search protocol based on PRISMA guidelines and PICO framework for the identification and selection of valid studies by using various databases like PubMed, Cochrane Library, and Embase. Where “*n*” is the number of studies at each step of the search strategy.

For the identification and selection of eligible studies, PRISMA guidelines were followed to define a search protocol systematically, as represented in Figure 1. The defined keywords and MeSH terms (*CYP1A1* mutation, MspI polymorphism, Ile462Val polymorphism, genetic variants, genetic predisposition to disease, cancer risk, cancer susceptibility, case–control, and association studies) were incorporated into the PubMed database and records were identified that include the studies comprehending the association of *CYP1A1* polymorphisms and cancer susceptibility. Before screening, 580 duplicate records, no ineligible records by automation tools, and 1520 records were published before 10 years. One thousand five hundred and seventy‐three records underwent screening of title, abstract, full‐text, and free full‐texts, from which only 533 records were excluded. Lastly, 1040 reports were assessed for eligibility, within which some were not accessible in full text (*n* = 95), meta‐analysis (*n* = 80), absence of genotype/allelic distribution (*n* = 313), and others did not meet the eligibility criteria (*n* = 532). The final eligible studies included in this meta‐analysis that met the inclusion and exclusion criteria were 20 (Amrani et al. 2016; Elderdery et al. 2024; Girdhar et al. 2016; Hoidy et al. 2019; Kleine et al. 2015; Li et al. 2016; Liu et al. 2016; Matos et al. 2016; Mukry et al. 2022; Nigam et al. 2019; Parada Jr. et al. 2017; Proença et al. 2015; Sakai et al. 2016; Salimi et al. 2016; Sharma et al. 2019; Singh and Ghosh 2019; Tan et al. 2016; Wongpratate et al. 2020; Xu and Tan 2024; Zhao et al. 2021), which comprehended the association of CYP1A1 polymorphs with cancer susceptibility.

### 3.2 Characteristics of the Included Studies

A total of 20 studies were included in this meta‐analysis, which contain different ethnicities and populations of individuals exhibiting *CYP1A1* mutations and polymorphisms (Amrani et al. 2016; Elderdery et al. 2024; Girdhar et al. 2016; Hoidy et al. 2019; Kleine et al. 2015; Li et al. 2016; Liu et al. 2016; Matos et al. 2016; Mukry et al. 2022; Nigam et al. 2019; Parada Jr. et al. 2017; Proença et al. 2015; Sakai et al. 2016; Salimi et al. 2016; Sharma et al. 2019; Singh and Ghosh 2019; Tan et al. 2016; Wongpratate et al. 2020; Xu and Tan 2024; Zhao et al. 2021). Characteristics of the included studies are represented in Table 1. The characteristic table includes author, year of the study, country, population/ethnicity, sample size, age of participants, cancer type, mutation, genotype polymorphisms, and genotyping methods utilized for the identification of particular *CYP1A1* polymorphisms. Furthermore, the total number (%) of participants based on their genotype and allelic distribution among case and control groups was also collected or extracted from all studies to perform statistical meta‐analysis. All studies were published within the last 10 years and follow the study design of case–control studies, meeting the eligibility criteria. However, the study participants exhibit different types of cancer associated with *CYP1A1* mutations and their genotypic polymorphisms. In most of the studies, the basic genotyping method performed was polymerase chain reaction‐restriction fragment length polymorphism (PCR‐RFLP). Almost 11 studies involved MspI polymorphisms of the *CYP1A1* genetic mutation (Girdhar et al. 2016; Kleine et al. 2015; Li et al. 2016; Liu et al. 2016; Matos et al. 2016; Nigam et al. 2019; Proença et al. 2015; Sharma et al. 2019; Singh and Ghosh 2019; Tan et al. 2016; Xu and Tan 2024), while the remaining nine studies involved Ile462Val polymorphism (Amrani et al. 2016; Elderdery et al. 2024; Hoidy et al. 2019; Mukry et al. 2022; Parada Jr. et al. 2017; Sakai et al. 2016; Salimi et al. 2016; Wongpratate et al. 2020; Zhao et al. 2021).

**TABLE 1:** Characteristics of the studies included in this meta‐analysis.

Author/year	Country	Study design	Population/ethnicity	Cancer type	Mutation	Genotype polymorphs	Genotyping method
Proença et al. (2015)	Brazil	Case–control	Brazilian	Sporadic colorectal cancer	CYP1A1	MspI	PCR‐RFLP
Kleine et al. (2015)	Brazil	Case–control	Brazilian	Cervical cancer	CYP1A1	MspI	PCR‐RFLP
Matos et al. (2016)	Portugal	Case–control	European	Cervical cancer	CYP1A1	MspI	PCR
Amrani et al. (2016)	Jordan	Multicenter Case–control	Jordanian	Breast carcinoma	CYP1A1	Ile462Val	PCR‐RFLP
Tan et al. (2016)	Malaysia	Case–control	3 major ethnic groups in Southeast Asia	Cervical carcinoma	CYP1A1	MspI	PCR‐RFLP
Sakai et al. (2016)	Bolivia	Case–control	Bolivians	Gallbladder cancer	CYP1A1	Ile462Val	PCR‐RFLP
Salimi et al. (2016)	Iran	Case–control	Iranian	Uterine Leiomyoma	CTP1A1	Ile462Val	PCR‐RFLP
Li et al. (2016)	China	Case–control	Chinese	Cervical cancer	CYP1A1	MspI	Genotyping assay
Girdhar et al. (2016)	India	Case–control	North Indian	Squamous cell lung cancer	CYP1A1	MspI	PCR‐RFLP
Liu et al. (2016)	China	Case–control	Chinese	Lung cancer	CYP1A1	MspI	PCR‐RFLP
Parada Jr. et al. (2017)	New York	Case–control	White	Breast cancer	CYP1A1	Ile462Val	Sequenom's high‐throughput MALDI‐TOF mass spectrometry
Singh and Ghosh (2019)	India	Population‐based case–control study	Manipuri, Naga, and Mizo	Nasopharyngeal cancer	CYP1A1	MspI (m1, T3801C)	PCR and Sanger sequencing
Nigam et al. (2019)	India	Case–control	North Indian	Oral pre‐cancer	CYP1A1	MspI	PCR‐RFLP
Sharma et al. (2019)	India	Case–control	North Indian	Epithelial ovarian cancer	CYP1A1	MspI (m1, T6235C)	PCR‐RFLP, AS‐PCR, and multiplexing
Hoidy et al. (2019)	Iraq	Population‐based case–control	Southern Iraq	Prostate cancer	CYP1A1	Ile462Val (rs1048943)	Alfa Gene
Wongpratate et al. (2020)	Thailand	Case–control	Northeast Thai	Cervical cancer	CYP1A1	Ile462Val	PCR‐RFLP
Zhao et al. (2021)	China	—	Chinese	Breast cancer	CYP1A1	Ile462Val	SNaPshot assay
Mukry et al. (2022)	Pakistan	Case–control	Karachi	Chronic myeloid leukemia	CYP1A1*2C	Ile462Val	Multiplex PCR approach
Xu and Tan (2024)	China	—	Chinese	Endometrial cancer	CYP1A1	MspI (rs4646903)	PCR‐RFLP
Elderdery et al. (2024)	Africa	Case–control	Sudanese	Chronic myeloid leukemia	CYP1A1*2C	Ile462Val (rs1048943)	PCR‐RFLP

All of the included studies exhibit different characteristics. Four studies were conducted in China, four in India, two in Brazil, and one each in Portugal, Jordan, Malaysia, Bolivia, Iran, New York, Thailand, Iraq, Pakistan, and Africa. These twenty studies include a total of 8867 participants distributed among the case/patient group (*n* = 3976) and control/healthy group (*n* = 4891). Based on specific cancer type, most of the studies focused on cervical cancer (*n* = 5), breast cancer (*n* = 3), lung cancer (*n* = 2), and chronic myeloid leukemia (*n* = 2), while others include colorectal cancer, gallbladder cancer, lung cancer, uterine leiomyoma, nasopharyngeal cancer, oral‐pre cancer, prostate cancer, epithelial ovarian cancer, and endometrial cancer. Moreover, the statistical data extracted from the studies for meta‐analysis are represented in Table 2.

**TABLE 2:** Genotype and allelic distribution of CYP1A1 polymorphs among case and control groups in numbers (%) to determine the odds ratio (OR).

Author/year	Ethnicity	Total sample (case/control)	Genotype distribution in Cases *n* (%)			Genotype distribution in controls *n* (%)			Allele distribution in cases *n* (%)		Allele distribution in controls *n* (%)	
			Wild	Heterozygote	Mutant	Wild	Heterozygote	Mutant	Wild	Mutant	Wild	Mutant
Proença et al. (2015)^a^	Brazilian	74/199	54 (73.0)	17 (23.0)	3 (4.0)	129 (64.8)	56 (28.1)	14 (7.0)	0.84	0.16	0.79	0.21
Kleine et al. (2015)	Brazilian	81/141	29.0 (35.8)	33.0 (40.7)	19.0 (23.5)	85.0 (60.3)	52.0 (36.9)	4.0 (2.8)	91.0 (56.2)	71.0 (43.8)	222.0 (78.7)	60.0 (21.3)
Matos et al. (2016)	Caucasian	105/124	64 (61.0)	36 (34.3)	5 (4.8)	99 (79.8)	23 (18.5)	2 (1.6)	164 (78.0)	46 (22.0)	221 (89.0)	27 (11.0)
Amrani et al. (2016)	Caucasian	112/115	102 (91.07)	10 (8.93)	0 (0)	102 (88.4)	12 (10.43)	1 (0.87)	0.956	0.044	0.944	0.056
Tan et al. (2016)	Asian	106/195	35 (33.0)	47 (44.3)	24 (22.6)	78 (40.0)	87 (44.6)	30 (15.4)	56 (51.9)	52 (48.1)	82 (63.1)	48 (36.9)
Sakai et al. (2016)	Others	32/86	1 (3.1)	20 (62.5)	11 (34.4)	10 (11.6)	33 (38.4)	43 (50.0)	22 (34.4)	42 (65.6)	53 (30.8)	119 (69.2)
Salimi et al. (2016)	Caucasian	105/112	70 (67)	35 (33)	0 (0)	80 (71.5)	32 (28.5)	0 (0)	175 (83)	35 (17)	192 (86)	32 (14)
Li et al. (2016)	Asian	360/368	188 (52.2)	145 (40.3)	27 (7.5)	227 (61.7)	128 (34.8)	13 (3.5)	521 (72.4)	199 (27.6)	582 (79.1)	154 (20.9)
Girdhar et al. (2016)	Caucasian	353/351	155 (43.9)	152 (43.05)	46 (13.03)	181 (51.5)	151 (43.0)	19 (5.4)	460 (65.3)	244 (34.7)	514 (73.2)	188 (26.8)
Liu et al. (2016)	Asian	308/253	99 (32.1)	145 (47.1)	64 (20.8)	113 (44.7)	111 (43.9)	29 (11.4)	—	—	—	—
Parada Jr. et al. (2017)	Caucasian	988/1021	873	60	4	890	92	3	—	—	—	—
Singh and Ghosh (2019)	Caucasian	123/189	50 (40.7)	52 (42.2)	21 (17.1)	89 (47.1)	69 (36.5)	31 (16.4)	—	—	—	—
Nigam et al. (2019)	Caucasian	250/250	104 (41.60)	110 (44.0)	36 (14.40)	106 (42.40)	112 (44.80)	32 (12.80)	319 (63.80)	181 (36.20)	324 (64.80)	176 (35.20)
Sharma et al. (2019)	Caucasian	100/100	26 (26)	56 (56)	18 (18)	34 (34)	52 (52)	14 (14)	—	—	—	—
Hoidy et al. (2019)	Caucasian	100/150	70 (70.0)	14 (14.0)	16 (16.0)	92 (61.3)	41 (27.3)	17 (11.3)	154 (77.0)	46 (23.0)	225 (75.0)	75 (25.0)
Wongpratate et al. (2020)	Others	204/204	96 (47.06)	90 (44.12)	18 (8.82)	99 (48.53)	90 (44.12)	15 (7.35)	0.691	0.309	0.706	0.294
Zhao et al. (2021)	Asian	140/140	80 (57.1)	55 (39.3)	5 (3.6)	100 (71.4)	31 (22.2)	9 (6.4)	—	—	—	—
Mukry et al. (2022)	Asian	99/169	63 (64)	15 (15)	21 (21)	137 (81)	15 (9)	17 (10)	—	—	—	—
Xu and Tan (2024)	Asian	310/624	167 (53.9)	123 (39.7)	20 (6.4)	364 (58.3)	232 (37.2)	28 (4.5)	457 (73.7)	163 (26.3)	960 (76.9)	288 (23.1)
Elderdery et al. (2024)	Others	26/100	6 (23.1)	14 (53.8)	6 (23.1)	37 (37)	55 (55)	8 (8)	26 (50)	26 (50)	129 (64.5)	71 (35.5)

^a^ Proença et al. (2015) stated genotype distribution counts combined for heterozygote and mutant, where the count (%) was calculated based on the total sample reported in the study. Minor discrepancies between individual genotype counts and reported totals may reflect methodology.

### 3.3 Quality Assessment of Included Studies

The quality of the studies was evaluated using the Newcastle‐Ottawa scale, whose total score ranges from 0 to 9, as summarized in Table 3. Each study was assessed using three domains of the Newcastle‐Ottawa scale (NOS) and assigned stars according to the information provided in that study. During assessment, two reviewers independently evaluated, assigned stars, and reviewed each study among domains to determine the total NOS score, while in case of any disagreements, a third reviewer resolved the issues arising among them. All of the studies included in this meta‐analysis were of high quality, except for eight that were of moderate quality, and the total NOS score was found to be between 5 and 9.

**TABLE 3:** Assessment of the quality of the included case–control studies using the Newcastle‐Ottawa scale.

Author/year	Selection (0–4 stars)	Comparability (0–2 stars)	Outcome (0–3 stars)	Total NOS (0–9)	Quality rating
Proença et al. (2015)	****	**	**	8	High quality
Kleine et al. (2015)	****	*	**	7	High quality
Matos et al. (2016)	**	**	***	7	High quality
Amrani et al. (2016)	***	*	***	7	High quality
Tan et al. (2016)	**	*	***	6	Moderate quality
Sakai et al. (2016)	**	*	***	6	Moderate quality
Salimi et al. (2016)	***	*	***	7	High quality
Li et al. (2016)	**	*	***	6	Moderate quality
Girdhar et al. (2016)	***	*	***	7	High quality
Liu et al. 2016	**	*	***	6	Moderate quality
Parada Jr. et al. (2017)	****	**	***	9	High quality
Singh and Ghosh (2019)	***	**	***	8	High quality
Nigam et al. (2019)	**	*	***	6	Moderate quality
Sharma et al. (2019)	***	*	***	7	High quality
Hoidy et al. (2019)	***	*	***	7	High quality
Wongpratate et al. (2020)	****	**	***	9	High quality
Zhao et al. (2021)	**	*	***	6	Moderate quality
Mukry et al. (2022)	*	*	***	5	Moderate quality
Xu and Tan (2024)	**	*	***	6	Moderate quality
Elderdery et al. (2024)	****	*	***	8	High quality

* Assigned for meeting a single criterion within the domain.

** Assigned for meeting two criteria.

*** Assigned for meeting three criteria.

**** Maximum number of stars awarded for that domain (all criteria met).

### 3.4 Subgroup Analysis for MspI Polymorphism

**FIGURE 2:**
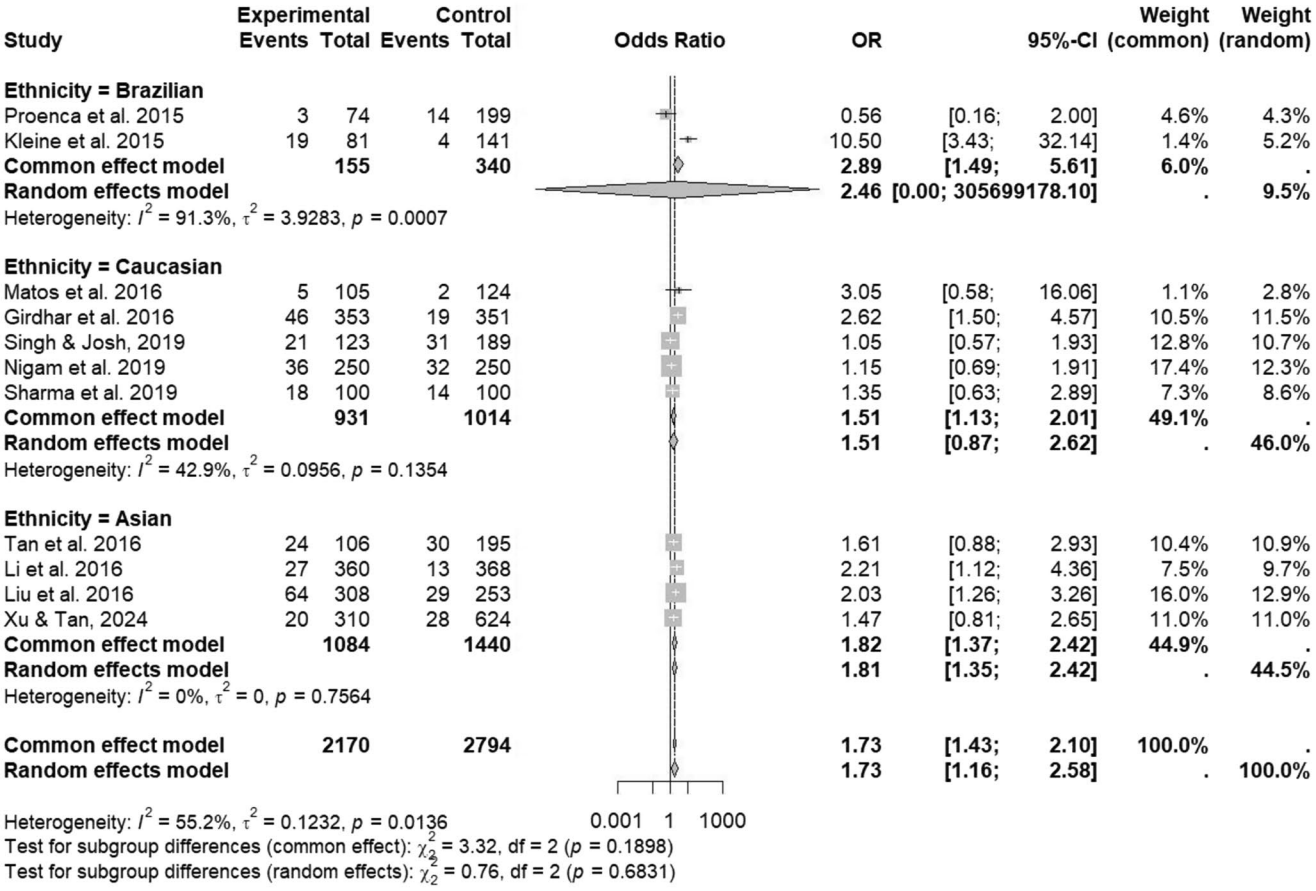
Forest plot of *CYP1A1* MspI polymorphism among three ethnic groups, such as Brazilian, Caucasian, and Asian populations.

In a subgroup analysis of *CYP1A1* MspI polymorphism, odds ratios with 95% CI were calculated among three different ethnic populations, such as Brazilian, Caucasian, and Asian. The association of cancer risk with MspI polymorphisms is represented in Figure 2. Pooled effect size calculated through the random‐effects model was 1.73 [1.16; 2.58] while the heterogeneity was 55.2%. Among these three ethnic groups, significant associations were found within Brazilian ethnicity with an OR of 2.46 [0.00; 305699178.1] and a significant *p*‐value of < 0.0001. Meanwhile, nonsignificant associations were found in Caucasian and Asian ethnicity, validating that *CYP1A1* MspI polymorphisms are not associated with cancer risk and susceptibility within these populations. However, a significant association was found within overall populations, but the strongest and most consistent association was in the Asian population, indicating ethnic‐specific genetic susceptibility. The observed heterogeneity among the Brazilian population limits the results of subgroup analysis, which arises due to differences in cancer types and patient demographics.

### 3.5 Subgroup Analysis for Ile426Val Polymorphism

**FIGURE 3:**
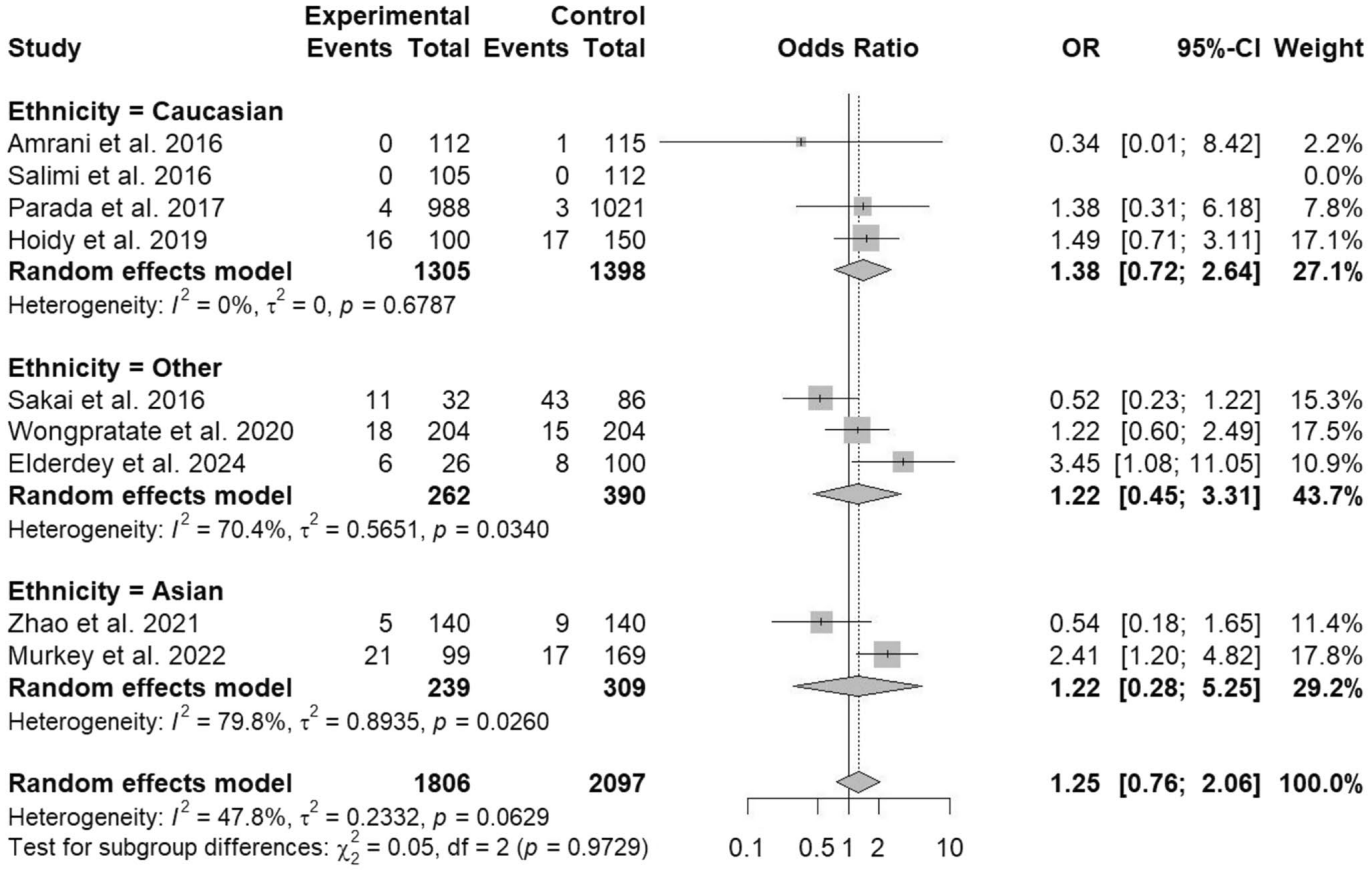
Forest plot of *CYP1A1* Ile462Val polymorphism among three ethnic groups, including Caucasian, Asian, and others.

The results of the subgroup analysis are shown in Figure 3. Subgroup analysis of *CYP1A1* Ile462Val polymorphism, pooled effect size calculated as OR was 1.25 with 95% CI [0.76; 2.06] and with a nonsignificant *p*‐value. Significant associations were found in individuals with Ile462Val polymorphism among the Asian ethnic group, with heterogeneity and small sample size among studies. Within Caucasian ethnicity, the observed OR is 1.38 with 95% CI [0.72; 2.64], *p*‐value of 0.6787, and heterogeneity of *I* ^2^ = 0%. Other ethnic groups also exhibit nonsignificant associations with an OR of 1.22 [0.45; 3.31], while the Asian group contains an OR of 1.22 [0.28; 5.25]. Other ethnic groups exhibit high heterogeneity and a nonsignificant *p*‐value, which affects the overall OR ratio and *p*‐value. All subgroup estimates had wide confidence intervals with notable heterogeneity in Asian and Other ethnic groups. These findings underscore the importance of considering ethnic diversity in genetic association studies related to cancer risk or susceptibility.

### 3.6 Publication Bias

**FIGURE 4:**
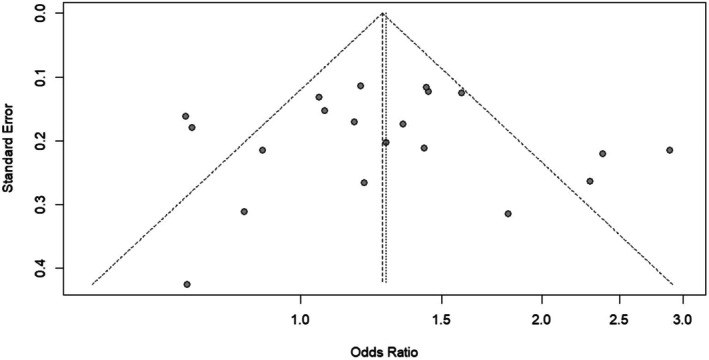
Funnel plot of all included studies to assess the publication bias by plotting OR on the *x*‐axis and standard error (SE) on the *y*‐axis, where each dot represents the study and the funnel represents the confidence interval of the overall effect size.

The funnel plot for the *CYP1A1* gene variant studies presents an approximate rough symmetrical arrangement of the different effect sizes around the line of pooled OR, suggesting the absence of significant publication bias (Figure 4). As predicted, the bigger studies are found in the upper part of the plot while the smaller ones are spread out in the lower part. The symmetry suggests that there was no significant publication bias among the studies that were included. The visual observation corresponds with the nonsignificant results of statistical tests by Begg and Egger (*p* > 0.05), hence confirming that the influence of small‐study effects or selective publication on the results of the meta‐analysis is unlikely to be substantial. Publication bias was assessed using statistical tests like Egger's and Begg's tests for all genetic models of the *CYP1A1* polymorphisms, as presented in Table 4. For the *CYP1A1* MspI polymorphism, publication bias assessment showed no significant bias for all genetic models. Under the allelic model (T vs. C), Egger's test (*t* = 0.78, df = 9, *p* = 0.456) and Begg's test (*z* = 0.86, *p* = 0.3918) were not significant. For the dominant model (TC + CC vs. TT), Egger's test (*t* = 0.84, df = 9, *p* = 0.4231) and Begg's test (*z* = 0.86, p = 0.3918) were not significant. The recessive model (CC vs. TC + TT) also showed no bias, Egger's test (*t* = 1.46, df = 9, *p* = 0.1774) and Begg's test (*z* = 0.54, *p* = 0.5858).

**TABLE 4:** Publication bias assessment of *CYP1A1* genotype polymorphisms (MspI and Ile462Val) among different genetic models, using Egger's and Begg's tests.

Polymorphism	Genetic model		Egger's test				Begg's test		
			Estimate	*t*	df	*p*	Estimate	*z*	*p*
MspI	Allelic	T vs. C	1.821	0.78	9	0.456	11.00	0.86	0.3918
	Dominant	TC + CC vs. TT	1.440	0.84	9	0.4231	11.00	0.86	0.3918
	Recessive	CC vs. TC + TT	2.539	1.46	9	0.1774	7.000	0.54	0.5858
Ile462Val	Allelic	A vs. G	1.116	0.51	7	0.6251	4.000	0.42	0.6767
	Dominant	AG + GG vs. AA	2.054	1.40	7	0.2035	12.00	1.25	0.2109
	Recessive	GG vs. AG + AA	−0.912	−0.62	6	0.5588	−6.000	−0.74	0.4579

Publication bias assessment for the *CYP1A1* Ile462Val polymorphism showed no significant bias for all genetic models. The allelic model (A vs. G) was not significant with Egger's test (*t* = 0.51, df = 7, *p* = 0.6251) and Begg's test (*z* = 0.42, *p* = 0.6767). For the dominant model (AG + GG vs. AA), Egger's test was borderline significant (*t* = 2.054, df = 7, *p* = 0.2035) but above the conventional significance threshold, and Begg's test was not significant (*z* = 1.25, *p* = 0.2109). The recessive model (GG vs. AG + AA) showed no bias with Egger's test (*t* = −0.62, df = 6, *p* = 0.5588) and Begg's test (*z* = −0.74, *p* = 0.4579). Therefore, overall publication bias assessment using both Egger's and Begg's tests showed no significant bias for both *CYP1A1* polymorphisms for all genetic models. All *p*‐values > 0.05, so the observed associations between *CYP1A1* polymorphisms and cancer risk are unlikely to be due to publication bias. This strengthens our meta‐analysis conclusions and suggests that the reported associations are real rather than a reporting bias in the literature.

## 4 Discussion

The comprehensive meta‐analysis encompasses statistical data from 20 eligible case–control studies with a substantial sample size of 3976 cases and 4891 controls, aimed at elucidating the association between *CYP1A1* gene/polymorphisms (MspI and Ile462Val) and cancer susceptibility across diverse ethnic populations. The findings of this meta‐analysis provide evidence regarding ethnic‐specific associations, highlighting the complex role of *CYP1A1* in cancer predisposition. Ethnic diversity in the frequencies of *CYP1A1* alleles might be the reason for the different levels of susceptibility such as the Ile462Val variant being found to a greater extent in Asian groups and this could perhaps corroborate the observed associations more firmly.

The results summary for this meta‐analysis was represented in Table 5. For MspI polymorphisms, the overall pooled effect size calculated using a random‐effects model indicates significant associations of MspI polymorphisms with cancer risk (OR = 1.73; 95% CI [1.16; 2.58]) and high heterogeneity (*I* ^2^ = 55.2%; *p* = 0.0136). However, this overall significance was primarily derived from the Brazilian ethnic group because of its highly significant association (PR = 2.46; 95% CI [0.00; 305699178.1]) with high heterogeneity (*I* ^2^ = 91.3%). Conversely, the Caucasian ethnic group showed a nonsignificant association (OR = 1.51; 95% CI [0.99; 2.28], *p* = 0.1354) along with moderate heterogeneity (*I* ^2^ = 0%), suggesting consistent and albeit nonsignificant results. Meanwhile, the funnel plot for MspI polymorphism suggested mild asymmetry that indicates the presence of potential publication bias or may be due to the effects of the small study, where studies with nonsignificant or negative findings might be underreported.

**TABLE 5:** Associations of *CYP1A1* polymorphisms across genetic models by ethnic‐specific groups.

Polymorphism	Genetic model		Ethnic group	OR (95% CI)	*p*	*I* ^2^ (%)
MspI	Allelic	T vs. C	Overall	1.37 [1.13; 1.66]	< 0.05	58.2
			Brazilian	1.45 [0.38; 5.55]	< 0.05	94.1
			Caucasian	1.32 [1.08; 1.61]	< 0.05	0.0
			Asian	1.38 [1.20; 1.58]	< 0.05	0.0
	Dominant	TC + CC vs. TT	Overall	1.39 [1.15; 1.68]	< 0.05	54.3
			Brazilian	1.35 [0.35; 5.24]	< 0.05	92.8
			Caucasian	1.38 [1.08; 1.76]	< 0.05	0.0
			Asian	1.40 [1.19; 1.66]	< 0.05	0.0
	Recessive	CC vs. TC + TT	Overall	1.69 [1.22; 2.34]	< 0.05	64.3
			Brazilian	2.71 [0.21; 25.08]	< 0.05	94.4
			Caucasian	1.51 [1.13; 2.01]	< 0.05	0.0
			Asian	1.81 [1.36; 2.41]	< 0.05	26.9
Ile462Val	Allelic	A vs. G	Overall	1.15 [0.87; 1.50]	0.33	53.8
			Caucasian	0.84 [0.66; 1.07]	0.16	0.0
			Asian	1.84 [1.11; 3.04]	< 0.05	79.8
			Others	1.14 [0.83; 1.57]	0.41	20.7
	Dominant	AG + GG vs. AA	Overall	1.20 [0.84; 1.71]	0.31	50.2
			Caucasian	0.78 [0.59; 1.03]	0.08	0.0
			Asian	2.11 [1.45; 3.06]	< 0.05	52.8
			Others	1.37 [0.76; 2.49]	0.30	46.1
	Recessive	GG vs. AG + AA	Overall	1.26 [0.77; 2.06]	0.36	47.8
			Caucasian	1.38 [0.72; 2.64]	0.68	0.0
			Asian	1.22 [0.28; 5.25]	0.79	79.2
			Others	1.16 [0.45; 2.99]	0.75	59.4

Regarding the Ile462Val polymorphisms, the stratification of studies into ethnic‐based subgroups yielded significant associations across some ethnic groups. For the Caucasian group, the association was nonsignificant (OR = 1.38; 95% CI [0.72; 2.64], *p* = 0.6787) with no heterogeneity (*I* ^2^ = 0%). The Asian group showed a somewhat significant association (OR = 1.22; 95% CI [0.28; 5.25], *p* = 0.0260), exhibiting high heterogeneity (*I* ^2^ = 79.8%). The wide confidence intervals observed across all subgroups of the Ile462Val polymorphism exhibit high heterogeneity and limit the significant findings of the study. While the results of funnel plots indicate significant superior symmetry, suggesting minimal publication bias. these findings collectively emphasize the critical importance of considering ethnic diversity in case–control or association studies about cancer risk.

Stratification analysis of *CYP1A1* polymorphisms based on ethnicity within genetic models (allelic, dominant, and recessive) was presented in Figures S1 and S2. For Ile462Val polymorphism of *CYP1A1*, overall meta‐analysis showed no significant association with cancer risk (OR = 1.15, 95% CI: 0.87–1.50) (Figure S1). However, ethnic heterogeneity was significant (*p* = 0.0174 for random effects). Under the allelic model (A vs. G), subgroup analysis by ethnicity showed no significant association in Caucasian (OR = 0.84, 95% CI: 0.66–1.07) and other ethnic groups (OR = 1.14, 95% CI: 0.83–1.57), but increased risk in Asian ethnicity (OR = 1.84, 95% CI: 1.11–3.04) (Figure S1a). Under the dominant model (AG + GG vs. AA), no overall significant association was found (OR = 1.20, 95% CI: 0.84–1.71) (Figure S1b). Stratification analyses suggested a protective effect approaching significance within Caucasian (OR = 0.78, 95% CI: 0.59–1.03), no significant association in others (OR = 1.37, 95% CI: 0.76–2.49), and significantly increased risk in the Asian subgroup (OR = 2.11, 95% CI: 1.45–3.06). Moreover, the recessive model (GG vs. AG + AA) showed no overall significant association (OR = 1.26, 95% CI: 0.77–2.06) (Figure S1c). Ethnic differences were not significant (*p* = 0.6086 for common effect; *p* = 0.9715 for random effects). Therefore, *CYP1A1* MspI showed consistent associations across multiple models and ethnic groups, with the strongest in the recessive model while *CYP1A1* Ile462Val showed ethnic specific associations with increased risk in Asian populations. Heterogeneity was observed in some analyses suggesting population specific factors may be involved.

In Figure S2, the allelic model (T vs. C) of the MspI polymorphism showed a significant overall association with cancer risk (OR = 1.37, 95% CI: 1.13–1.66) (Figure S2). Subgroup analysis of MspI polymorphisms based on ethnicity suggests a significant association in Brazilian (OR = 1.45, 95% CI: 0.38–5.55) while significantly increased risk was observed in Caucasian (OR = 1.32, 95% CI: 1.08–1.61) and Asian (OR = 1.38, 95% CI: 1.20–1.58) subgroups (Figure S2a). Under the dominant model (TC + CC vs. TT), the overall association was significant (OR = 1.39, 95% CI: 1.15–1.68); however, ethnic groups indicated distinct trends of cancer susceptibility in Brazilian (OR = 1.35, 95% CI: 0.35–5.24), Caucasian (OR = 1.38, 95% CI: 1.08–1.76), and Asian (OR = 1.40, 95% CI: 1.19–1.66) ethnic subgroups (S2b). Meanwhile, the recessive model (CC vs. TC + TT) showed a significant association with cancer risk (OR = 1.69, 95% CI: 1.22–2.34) (Figure S2c). Subgroup analysis showed a nonsignificant wide confidence interval in Brazilian (OR = 2.71, 95% CI: 0.21–35.08) and significantly increased risk in Caucasian (OR = 1.51, 95% CI: 1.13–2.01) or Asian (OR = 1.81, 95% CI: 1.36–2.41).

The observed associations, like significant findings for the MspI polymorphisms in the Brazilian group and consistent trends in Asian populations, are biologically plausible given the established role of the *CYP1A1* gene and its encoded enzyme. *CYP1A1* is a crucial Phase I metabolizing enzyme involved in both the activation and detoxification of various carcinogens. The MspI polymorphism (rs4646903) and Ile462Val polymorphism (rs1048943) are known to alter *CYP1A1* gene expression and enzymatic activity. For instance, the Ile462Val genetic variant is found to be associated with increased catalytic activity, which could lead to a more rapid conversion of pro‐carcinogens into reactive intermediates. Similarly, the MspI variant located in the 3′‐untranslated region may influence mRNA stability and translation efficiency, impacting enzyme levels.

The biological relevance to cancer development stems from the balance between carcinogen activation and detoxification. If a particular *CYP1A1* genotype leads to an overactive enzyme that predominately favors carcinogen activation over detoxification, individuals carrying such genotypes may have an increased accumulation of DNA‐damaging metabolites, which results in an elevation of their cancer risk. Conversely, genotypes that enhance detoxification or reduce activation could confer a protective effect. The population‐specific genetic architecture is characterized by varying allele frequencies of these polymorphisms across different ethnic groups.

The findings of this study present both confirmations and discrepancies when compared to individual studies and previous meta‐analyses on *CYP1A1* polymorphisms and cancer risk. The overall nonsignificant association for Ile462Val polymorphism in our meta‐analysis aligns with some individual studies that also reported no significant association between this variant and overall cancer susceptibility. However, the significant overall association for MspI polymorphism contrasts with some studies that found no association in other ethnic groups. The discrepancies observed across ethnic‐specific associations emphasize the significance of population stratification. Previous studies often pooled diverse ethnic groups, potentially masking true associations or introducing heterogeneity. This stratified analysis also addresses the limitations found in previous studies, revealing that the impact of *CYP1A1* polymorphisms on cancer susceptibility is not uniform across all populations. The high heterogeneity observed in certain subgroups (MspI in Brazilian, Ile462Val in Asian, and Others) suggests that factors beyond the polymorphism itself, such as specific cancer types included in those subgroups or unmeasured environmental cofactors, might be influencing results.

However, this meta‐analysis boasts several notable strengths that enhance the robustness and generalizability of its findings. The potential strengths of this study include a comprehensive search strategy, a large combined sample size, ethnic stratification, and a rigorous methodological approach. Despite its strengths, this meta‐analysis also exhibits several limitations that warrant consideration when interpreting the results. A primary concern is the heterogeneity observed between studies. While a random effects model was used to account for this, the presence of substantial heterogeneity suggests that factors beyond the investigated polymorphisms, such as differences in cancer types included and mild asymmetry, suggest the indication of publication bias. This can be due to the limited availability of data found in some specific subgroups. Analysis was based on unadjusted estimates from the included studies, which require a more precise analysis for comprehensive adjustment of these covariates.

Future research should elucidate the complex interplay of *CYP1A1* polymorphisms and cancer susceptibility regarding specific cancer types. Large‐scale population‐based studies should be specifically designed to investigate the association of *CYP1A1* variants with specific cancer types within well‐defined ethnic populations. Such studies contribute to a large sample size and statistical analysis to determine more subtle genetic effects, ultimately reducing the impact of heterogeneity. Moreover, functional studies comprehending gene–environment interactions and polygenic risk scores (PRS) that incorporate *CYP1A1* polymorphisms alongside other established genetic risk factors for specific cancers hold significant promise. A PRS approach can integrate the cumulative effect of multiple low‐penetrance variants, potentially offering a more accurate and robust tool for cancer risk prediction and stratification than single‐SNP analysis.

## 5 Conclusion

The findings of this meta‐analysis summarize *CYP1A1* polymorphisms (MspI and Ile462Val) and cancer risk across various ethnic groups and show different population‐specific associations. The *CYP1A1* Ile462Val polymorphism has ethnic‐specific effects with increased cancer risk confined to Asian populations, while the MspI polymorphism has consistent and significant associations with increased cancer risk across multiple ethnic groups and genetic models, with the strongest effect under the recessive model (OR = 1.69, 95% CI: 1.22–2.34). Publication bias assessment showed no significant bias across all genetic models. These results indicate that *CYP1A1* polymorphism effects on cancer risk are complex and population‐dependent; MspI has broader ethnic associations than Ile462Val, so an ethnicity‐stratified approach is important in genetic association studies and population‐specific studies are needed to understand *CYP1A1*'s role in cancer genetics.

## Author Contributions

A.Y. and N.U.K. did the literature search, extracted the data, did the analysis, and wrote the manuscript. A.Y., N.U.K. and A.U. oversaw the data extraction and review of the final manuscript.

## Funding

The authors have nothing to report.

## Ethics Statement

The authors have nothing to report.

## Consent

The authors have nothing to report.

## Conflicts of Interest

The authors declare no conflicts of interest.

## Supporting information


**Figure S1:** Stratification analyses by ethnicity between CYP1A1 MspI polymorphisms and cancer susceptibility within (a) allelic model (T vs. C), (b) dominant model (TC + CC vs. TT), and (c) recessive model (CC vs. TC + TT). Where the square and the horizontal lines represented the OR (95% CI), the diamond represents the overall effect size, and the area/size of the square indicated the weight of the study using the random‐effects model
**Figure S2:** Stratification analyses by ethnicity between CYP1A1 Ile462Val polymorphisms and cancer susceptibility within (a) allelic model (A vs. G), (b) dominant model (AG + GG vs. AA), and (c) recessive model (GG vs. AG + AA). Where the square and the horizontal lines represented the OR (95% CI), the diamond represents the overall effect size, and the area/size of the square indicated the weight of the study using the random‐effects model.

## Data Availability

The data that support the findings of this study are available from the corresponding author upon reasonable request.
