# Supplementary material for: Meta‐Analysis of CYP1A1 MspI and Ile462Val Polymorphisms in Cancer Susceptibility Among Different Ethnic Populations
Source: Environ Mol Mutagen. 2025 Dec 10;67(1-2):e70042. doi: 10.1002/em.70042 (PMC13063359; doi:10.1002/em.70042)

**Supplementary Figures:**

**Figure S1:** Stratification analyses by ethnicity between CYP1A1 MspI polymorphisms and cancer susceptibility within (a) allelic model (T vs C), (b) dominant model (TC + CC vs TT), and (c) recessive model (CC vs TC + TT). Where the square and the horizontal lines represented the OR (95% CI), the diamond represents the overall effect size, and the area/size of the square indicated the weight of the study using the random-effects model.

**(a)**

**
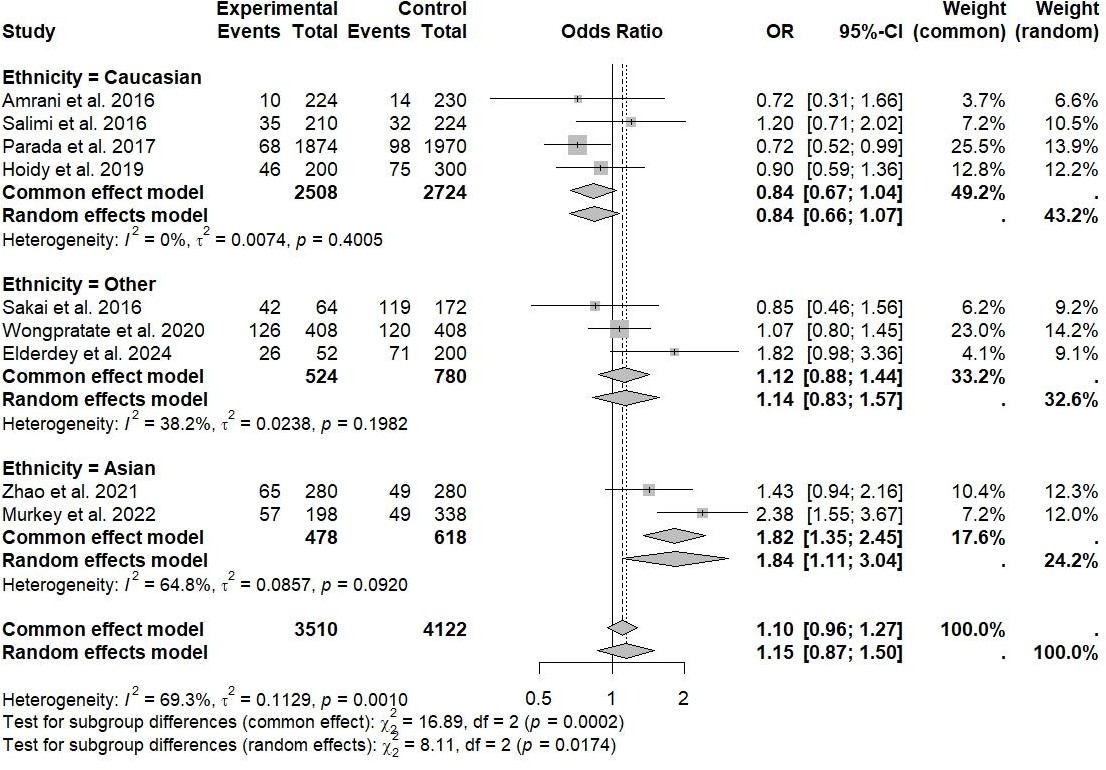
**

**(b)**

**
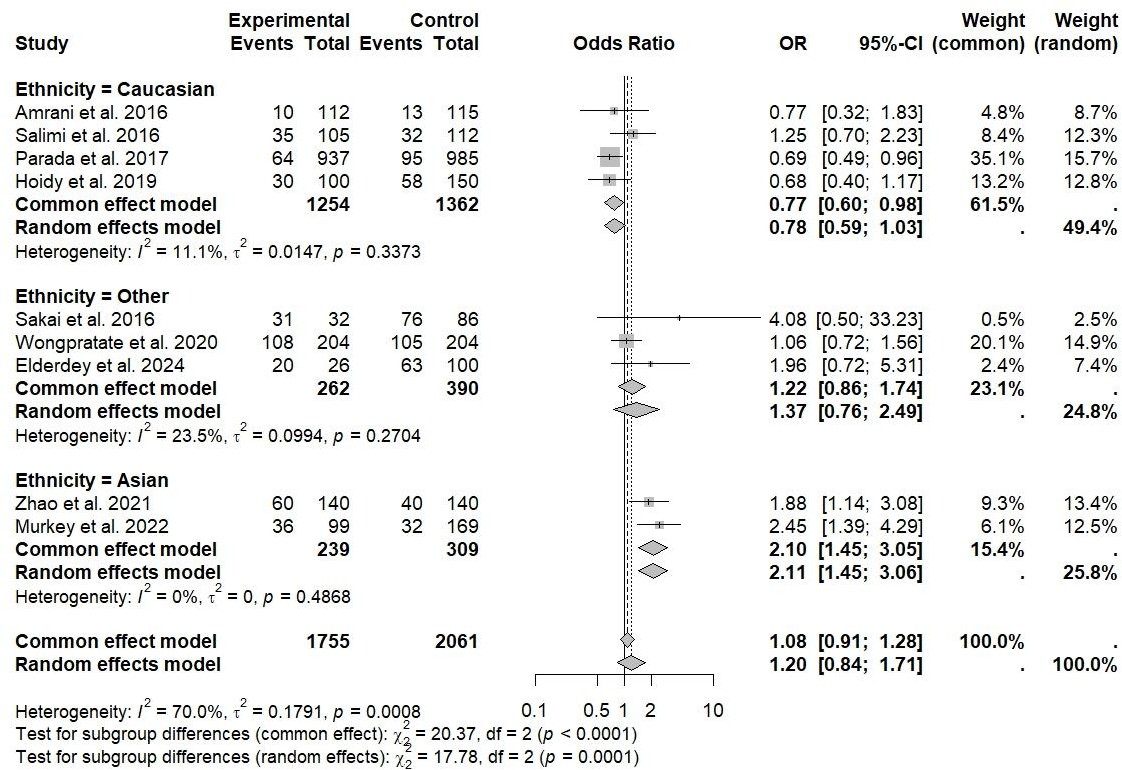
**

**(c)**


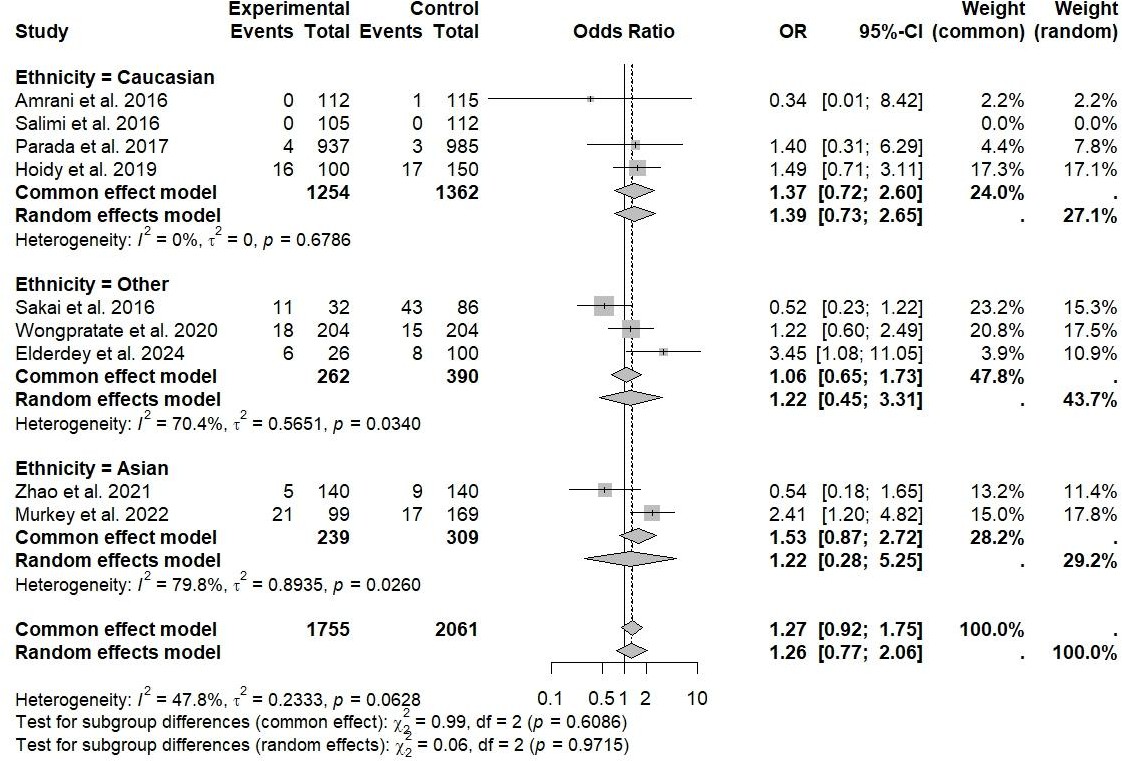


**Figure S2:** Stratification analyses by ethnicity between CYP1A1 Ile462Val polymorphisms and cancer susceptibility within (a) allelic model (A vs G), (b) dominant model (AG + GG vs AA), and (c) recessive model (GG vs AG + AA). Where the square and the horizontal lines represented the OR (95% CI), the diamond represents the overall effect size, and the area/size of the square indicated the weight of the study using the random-effects model.

**(a)**

**
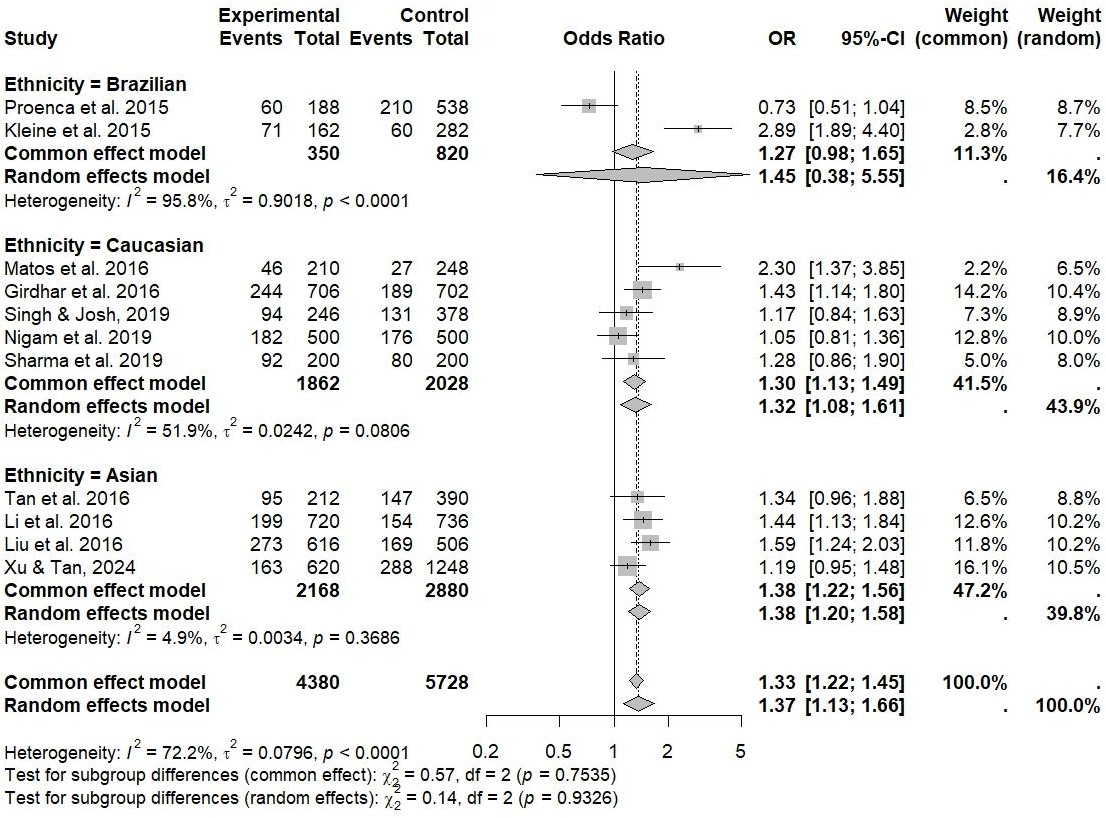
**

**(b)**

**
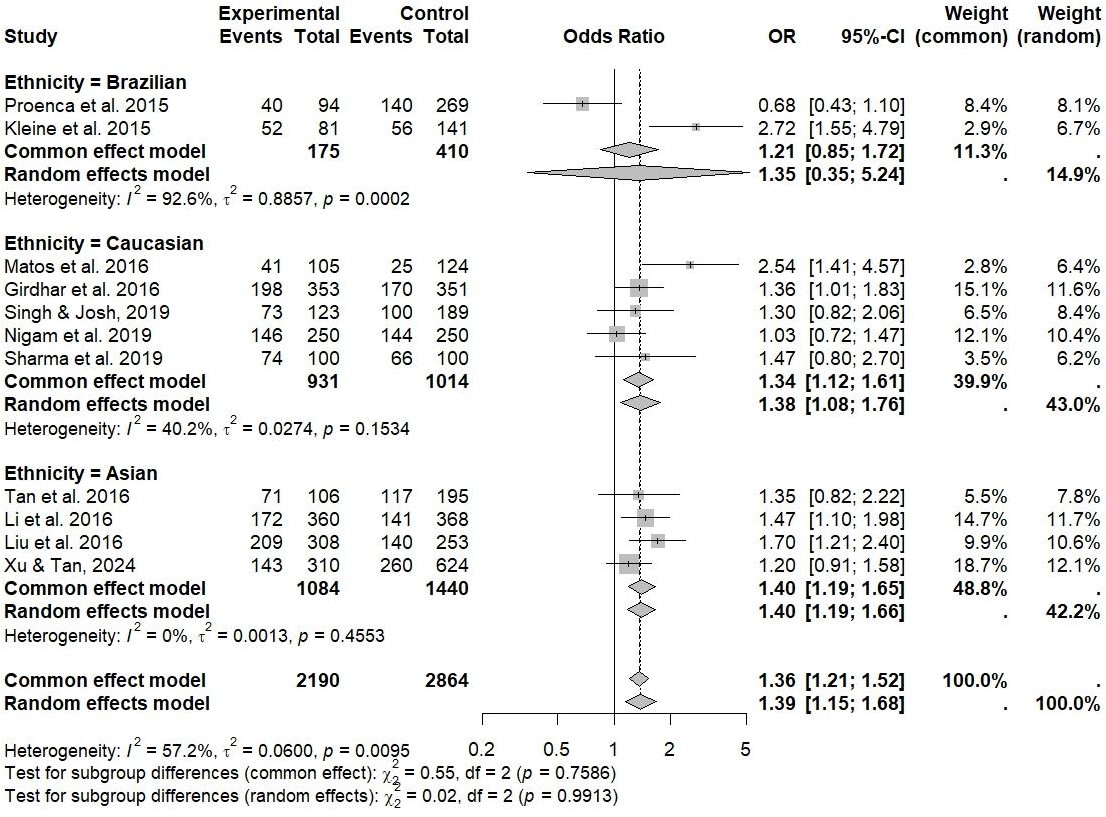
**

**(c)**


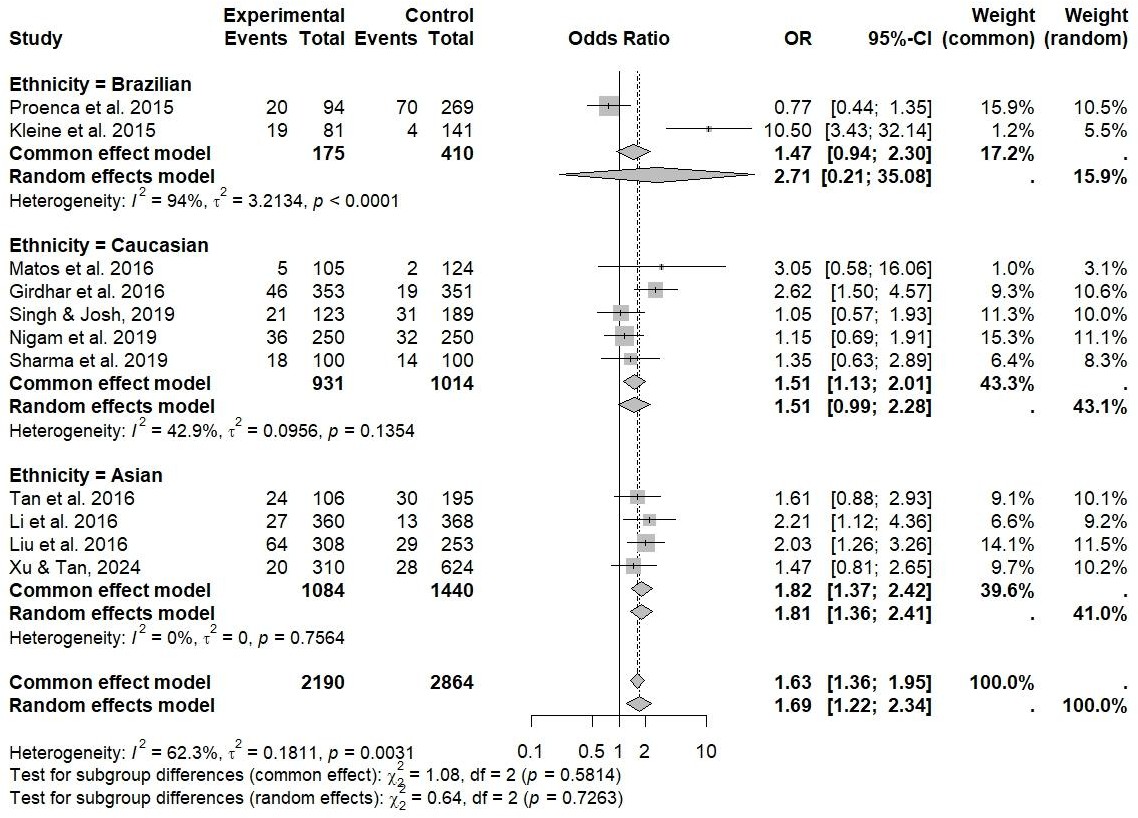

Supplement: Supplementary file 1 — Figure S1: Stratification analyses by ethnicity between CYP1A1 MspI polymorphisms and cancer susceptibility within (a) allelic model (T vs. C), (b) dominant model (TC + CC vs. TT), and (c) recessive model (CC vs. TC + TT). Where the square and the horizontal lines represented the OR (95% CI), the diamond represents the overall effect size, and the area/size of the square indicated the weight of the study using the random‐effects model Figure S2: Stratification analyses by ethnicity between CYP1A1 Ile462Val polymorphisms and cancer susceptibility within (a) allelic model (A vs. G), (b) dominant model (AG + GG vs. AA), and (c) recessive model (GG vs. AG + AA). Where the square and the horizontal lines represented the OR (95% CI), the diamond represents the overall effect size, and the area/size of the square indicated the weight of the study using the random‐effects model. [file EM-67-0-s001.docx]
